# Development of human hip joint in the second and the third trimester of pregnancy; a cadaveric study

**DOI:** 10.1186/1471-213X-13-19

**Published:** 2013-05-07

**Authors:** Adrian Masłoń, Marcin Sibiński, Mirosław Topol, Karol Krajewski, Andrzej Grzegorzewski

**Affiliations:** 1 Clinic of Orthopaedics and Paediatric Orthopaedics, Medical University of Lodz, Lodz, Poland; 2 Department of Angiology, Chair of Anatomy, Medical University of Łódź, Lodz, Poland

**Keywords:** Fetus, Hip joint development, Hip dysplasia, Hip anatomy

## Abstract

**Background:**

The purpose of the study was an evaluation of fetal hip joint morphology during the second and the third trimester of pregnancy. Serial sections were performed on 23 cadaver infants.

**Results:**

The mean lunar age was 6.6 months. Femoral shaft length (FSL) and width of the proximal and distal epiphysis were x-rayed to determine fetal age. The neck shaft angle (NSA), the femoral antetorsion angle (FAA), the acetabulum anteversion angle (AAA) and the acetabulum slope angle (ASA) were measured. Hip development ratios were plotted for all cadaveric species and revealed: flat FSL/NSA slope pattern, upward FSL/FAA slope pattern and downward slope pattern for FSL/ASA and FSL/AAA ratios. The changes, observed during the developmental period, were not statistically significant. NSA did not change during the second or the third pregnancy trimester. FAA increased during pregnancy but the changes were not statistically significant. AA, as well as ASA, showed a decreasing trend during the second and the third pregnancy trimester, however, with no correlations to age.

**Conclusion:**

Despite an increasing depth and growing dimensions of the acetabulum in the uterus, its orientation does not change in any significant way.

## Background

There have been a few reports about embryological hip development during early stages of the intrauterine life period [1-3], however, the development of hip joint alone in the second and the third trimester of gestation has rather been poorly described, thus deserving much more attention of researchers. The actual knowledge is, in general, not exhaustive as it is, by and large, gained from animal experiments, MRI scans and radiograph analyses [2,4].

The aim of the reported study was to determine fetal hip joint morphology during the second and third trimesters of pregnancy.

## Methods

The study had been approved by the Bioethical Committee of the Medical University in Lodz. An informed consent was obtained from involved individuals for the storage and use of their foetuses for research purposes.

**Figure 1:**
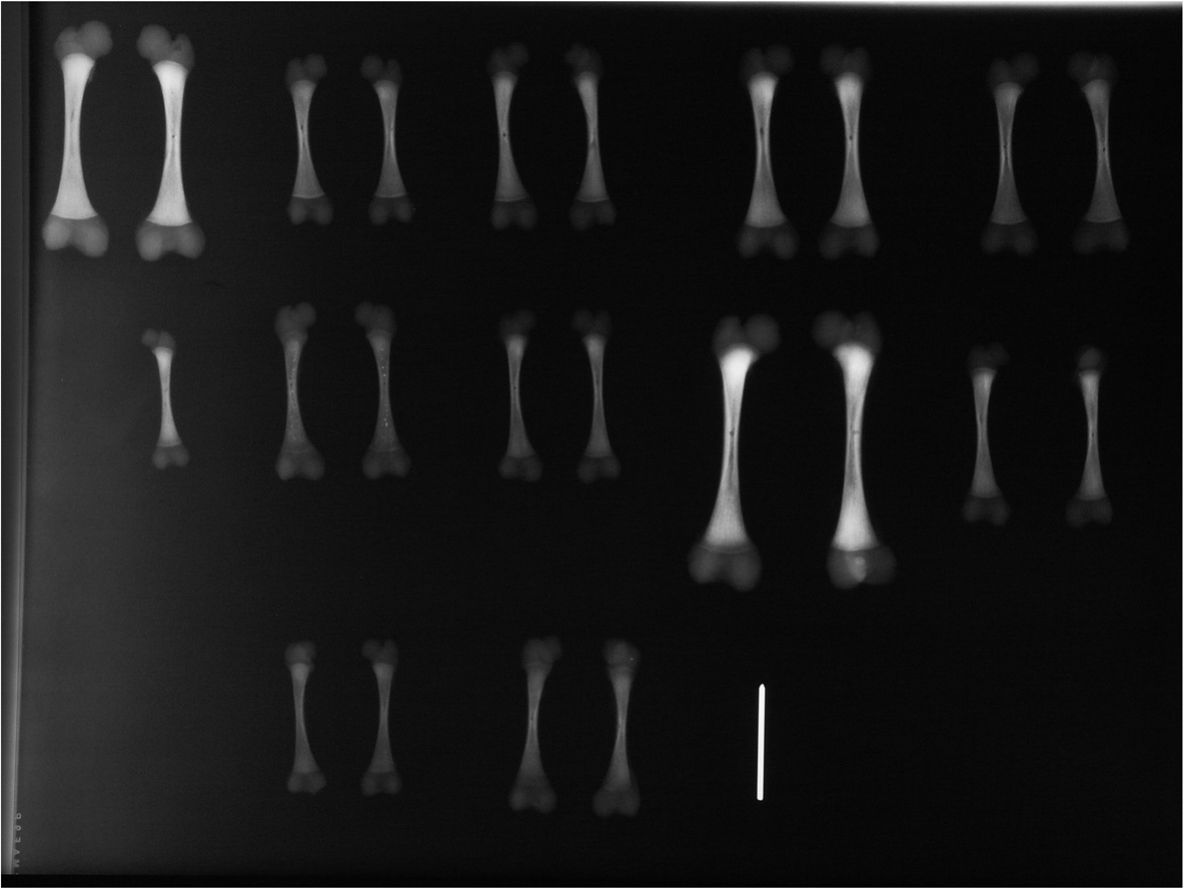
Femur radiographs were used to determine femoral shaft length (FSL) and fetal age.

**Figure 2:**
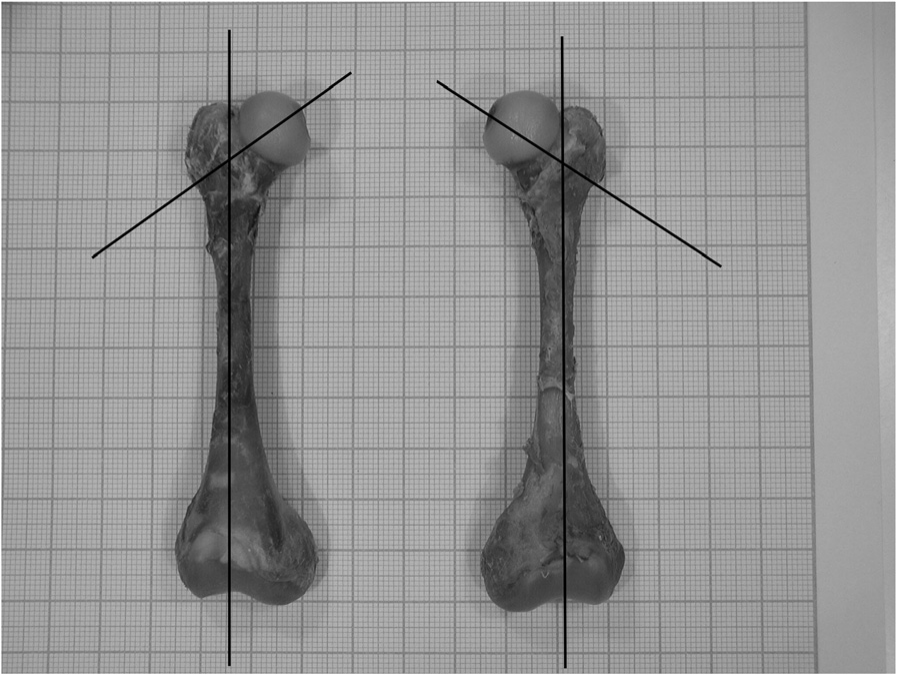
Neck shaft angle (NSA), measured on photos of cadaver femurs with support of the Gimp computer program.

**Figure 3:**
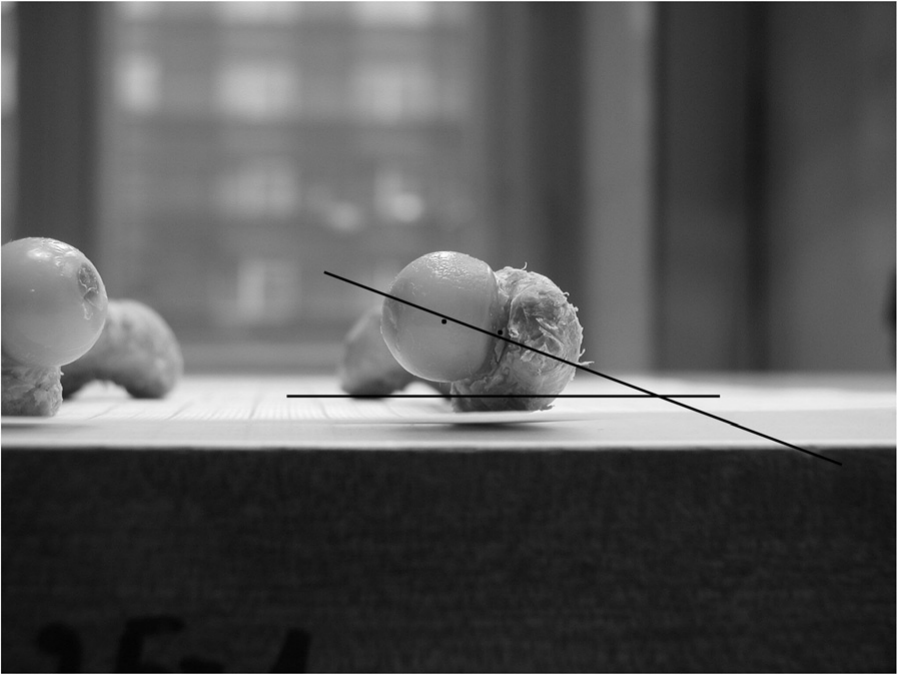
Femoral antetorsion angle (FAA), measured on photos of cadaver femurs with support of the Gimp computer program.

**Figure 4:**
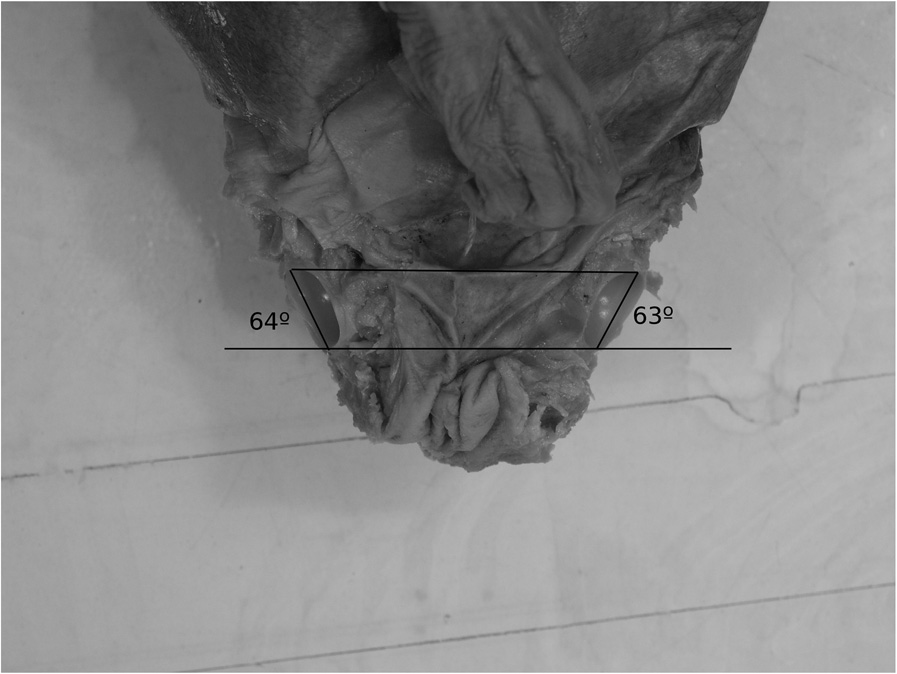
Acetabulum slope angle (ASA), measured on photos of cadaver femurs with support of the Gimp computer program.

In order to evaluate hip joint development throughout fetal stages, histological, serial sections were performed on 24 cadaver fetuses (48 hips), aged five to ten lunar months, including 14 female and ten male specimens. Formalin-fixed fetal specimens were property of the Department of Anatomy, Medical University of Lodz. No obstetric history was available in any of the cases. The left and the right femoral shaft length, the neck shaft angle, the acetabulum anteversion angle and the acetabulum slope angle were measured on both sides (left and right) of the body and the obtained values of each parameter on one side were compared with values of its counterpart on the other side. An error ≤ 4 degrees was accepted. If the difference between the left and the right side exceeded 4 degrees, such a sample was rejected for assumed evidence of hip dysplasia. One fetus was withdrawn from further research because of a difference in the acetabulum slope angle (ten degrees) and neck shaft angle (six degrees), identified between the left and right side. A statistical analysis was performed, using arithmetic means of left- and right-side results (23 fetuses). Femoral shaft length (FSL) was x-rayed to determine fetal age [5] [Figure 1]. The neck shaft angle (NSA) [Figure 2], the femoral antetorsion angle (FAA) [Figure 3], the acetabulum anteversion angle (AAA) and the acetabulum slope angle (ASA) were measured [see Figure 4]. NSA, FAA and ASA measurements were obtained from photos of cadaver femur and pelvis specimens. In order to standardize the pelvis position, a K wire was inserted through the obturator foramen, next to the anterior obturator tubercle and on the obturator crest of the superior ramus of the pubis. A studied femur was positioned on a table in a regular manner to identify femoral version with both femur condyles, placed on a flat surface. The AAA angle was measured directly on fetal pelvis. Fetal pelvis was laid on two posterior superior iliac spines and fixed to table. Next, AAA was measured between the line, perpendicular to the base (table surface) and the K-wire, running in the middle of the acetabulum, through the posterior and anterior rim. Finally, in order to assess the pre-natal development of human hip joint, FSL/NSA, FSL/FAA, FSL/ASA and FSL/AAA ratios were calculated. Spearman's rank correlation coefficient was used for statistical analysis.

## Results

The mean lunar age was 6.6 months, ranging from five to ten months [see Table 1].

**Table 1:** Femoral shaft length, proximal and distal epiphysis width and calculated lunar age of the studied fetuses

**Specimen number**	**Femoral shaft length**	**Proximal epiphysis width**	**Distal epiphysis width**	**Age (lunar months)**	**Sex**
1	57.4	12.0	16.2	8.5	m
2	40.6	8.8	11.4	6	f
3	44.9	8.8	11.7	6.5	f
4	53.8	10.9	14.6	8	m
5	49.5	10.3	12.7	7.5	m
6	34.7	6.7	8.3	5.5	m
7	41.9	9.0	11.2	6	m
8	42.6	8.2	10.0	6.5	f
9	73.2	15.0	17.7	10	m
10	45.0	8.3	11.4	6.5	f
11	37.9	6.1	9.3	5.5	m
12	43.2	8.6	11.0	6.5	m
13	53.4	10.5	14.3	7.5	f
14	37.9	7.2	8.4	5.5	m
15	51.3	10.1	14.6	7.5	m
16	45.6	9.0	13.1	6.5	m
17	35.0	6.2	7.5	5.5	f
18	41.6	7.5	10.3	6	f
19	45.4	9.6	13.1	6.5	f
20	30.1	5.1	7.9	5	m
21	44.3	7.9	11.9	6.5	f
22	31.0	6.9	8.4	5	f
23	52.0	8.4	12.2	7	m

Hip development ratios were plotted for all cadaveric samples. NSA values did not show any significant changes in time, being, on the average, at 119.8, SD 26.2. The slope of FSL vs. NSA was −0.628, R^2^ = 0.0091. The correlation index was −0.095, demonstrating a flay slope pattern.

The FAA ranged from 15 to 33 degrees. The slope of FSL vs. FAA was 0.197, R^2^ = 0.13. The correlation index was 0.36, revealing an upward slope pattern. The AAA ranged from six to 35 degrees. The slope of FSL vs. AAA was 0.3966, R^2^ = 151. The correlation index was −0.133, demonstrating a downward slope pattern. The ASA ranged from 51 to 85 degrees with the slope of FSL vs. ASA at 0.397, R^2^ = 0.152. The correlation index was 0.388, demonstrating a downward slope pattern.

**Figure 5:**
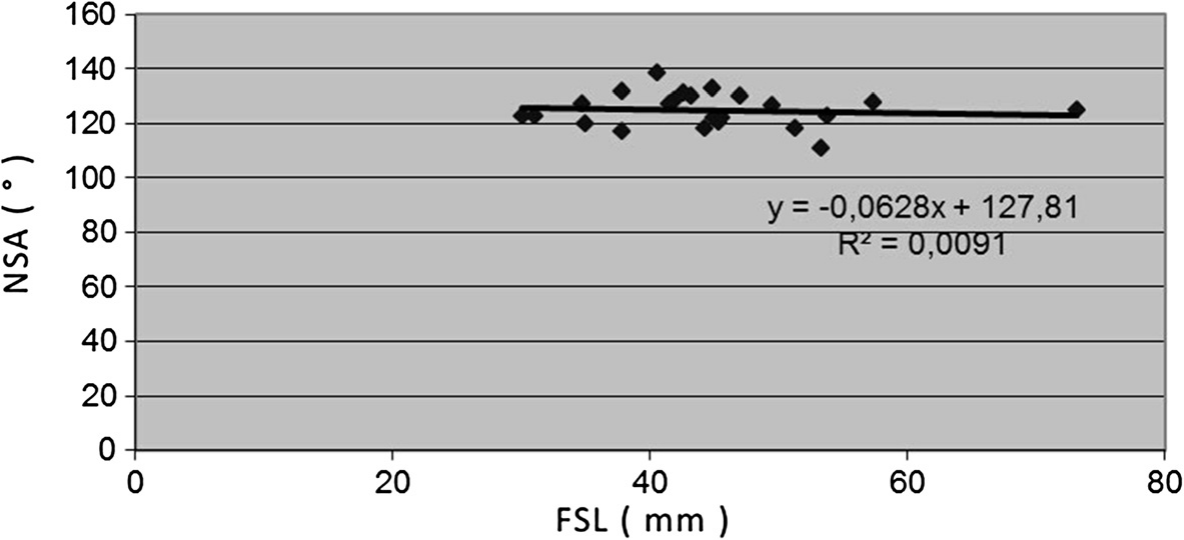
Correlations between age-determining femoral shaft length (FSL) and neck shaft angle (NSA).

**Figure 6:**
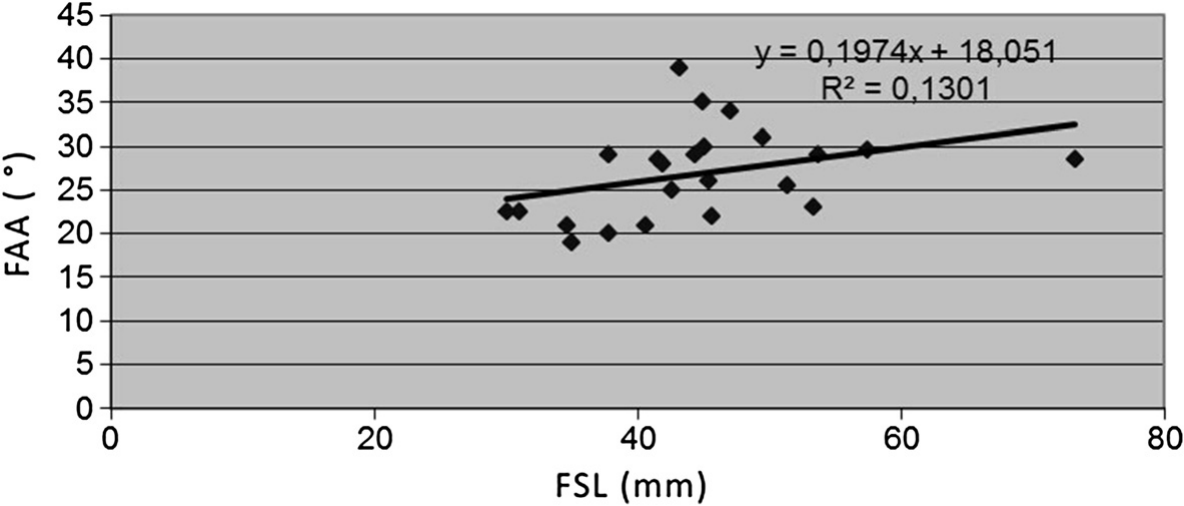
Correlations between age-determining femoral shaft length (FSL) and femoral antetorsion angle (FAA).

**Figure 7:**
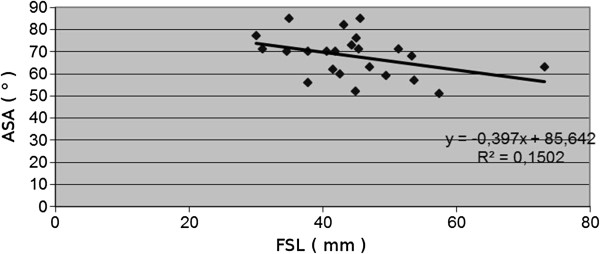
Correlations between age-determining femoral shaft length (FSL) and acetabulum slope angle (ASA).

**Figure 8:**
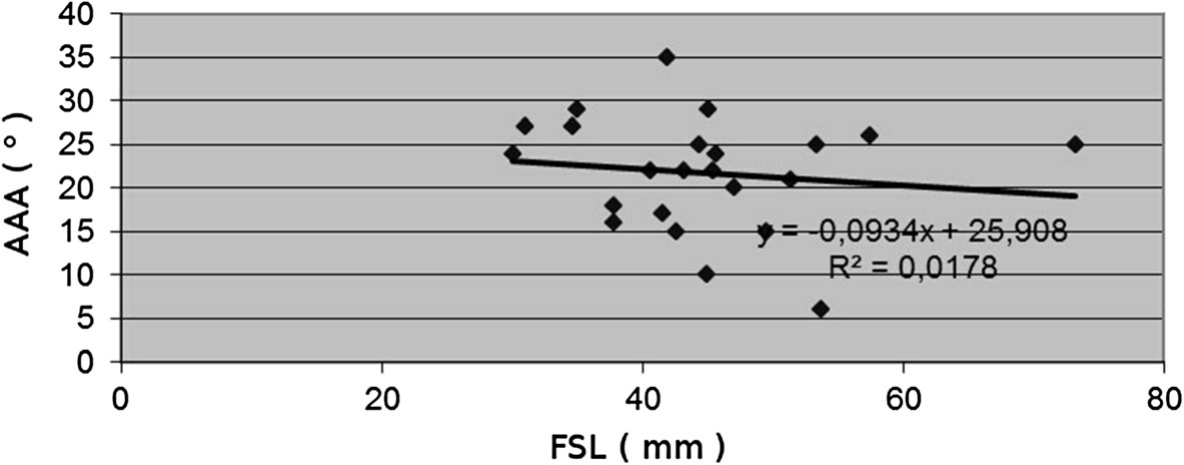
Correlations between age-determining femoral shaft length (FSL) and acetabulum anteversion angle (AAA).

The changes, observed during developmental period, were not statistically significant. [see Figures 5, 6, 7 and 8].

## Discussion

Decreased antetorsion and cervicodiaphyseal angles in the femoral neck, observed from birth till the end of growth process, have – thus far – been subject of many studies [6-8]. It has been demonstrated in frontal and horizontal planes that an inclination and anteversion of the capital epiphyseal plate position make it perpendicular to the resulting force, applied to hip in walking [6], thus reducing both these angles. Obesity [9], cerebral palsy (muscle imbalance) [10] or destruction of the abduction muscles during surgery [11] may change physiological forces, which are normally transferred through the joint, leading to pathological deformation of the proximal femur.

In our study, FAA increased in the second and the third trimester of pregnancy, being thus suggestive of mechanical stress that could have remodelled the proximal femur. The, so-called, human or fetal position of both lower limbs *in utero* imposes a relatively perpendicular position onto the acetabulum anatomical axis and the femoral neck. The femur is upside down and the femoral head points backwards, due to the anteversion angle. In this position, any external rotation of the femur will increase FAA. Furthermore, with the time passing by, the fetus has less and less space available in the uterus and its legs are gradually being pushed toward the trunk. This could explain the positive correlation between age and FAA. In opposition to FAA angle, NSA is more stable, not changing significantly, either during the second or the third trimester of pregnancy.

Jouve at al. also found via anatomical studies that the anteversion angle increased during the second half of gestation [12]. He drove a speculation that those changes may have been caused by mechanical stresses. Regarding the period before the 24^th^ week of intrauterine life, no conclusion could be drawn because of possible, size-related confounders. Neither any conclusion could be drawn, concerning NSA [12]. In the study of Walker and Goldsmith, the measured femoral angles demonstrated only a weak correlation with other hip variables and none of those angles appeared to be a useful indicator of normal joint development. Their observations indicate that soft tissue structures around the joint must play an important role in neonatal joint stability [13].

Following previously assumed theories and performed animal studies, Ralis and McKibbin confirmed, on the basis of their own cadaveric studies, that it was the shallow acetabulum, which was mostly responsible for neonatal hip instability It is well known that, in early gestational stages, the femoral head is accurately covered by the acetabulum. Later on, in gestational development, the proportion of the femoral head, covered by the acetabulum, decreases to its minimum, while increasing again after birth [14]. A further work by Walker and Goldsmith confirmed that the acetabular depth was the slowest growing variable in the perinatal period [13]. Lee at al. found that acetabular anteversion and femoral head coverage did not change significantly in early fetal stages (six to 20 weeks) [1]. Withby et al., being supported by postmortem MRI studies (gestation age between 17 and 42 weeks), identified some development in the acetabular dimension till the 20^th^ week, when growth demonstrated an exponential rate [2].

### Limitations of the study

The results of performed measurements are highly variable, what hampers any statistically significant conclusions with such relatively low patient numbers. A precise foetal age was unknown, being merely assumed from x-rayed femur length. Direct measurements on fetal specimens were affected by some error, resulting from small sizes of measured organs.

## Conclusions

The observed changes in the acetabulum position were not as dynamic as those in the proximal part of the femur. Our study focused on AAA, as well as on ASA changes in the second and the third trimester of pregnancy, demonstrating that only mild changes of those angles did not correlate with age during the period. In consequence, it may be speculated that neither the pelvis nor the acetabulum changes its position as much as the femur, thus allowing to regard the above-mentioned angles as relatively unchanged. On the basis of both previous studies and our observations, a conclusion may be drawn that, despite overtly increasing depth and dimensions of the acetabulum in the uterus, its orientation does not change in any significant way.

## Consent

The study had been approved by the Bioethical Committee of the Medical University of Łódź, Poland. An informed consent was obtained from individuals for storage and use of their foetuses for research purposes.

## Competing interests

The authors declare that they have no competing interests.

## Authors’ contributions

AM, MT, AG designed the experiments and acquired the data. AM,MT,MS,KK – performed pathological studies. AM, MS, AG analyzed the data and performed statistical analyses. AM,MS,MT,KK,AG contributed to interpretation of data. AM,MS drafted the manuscript. AM,MS,MT,KK,AG revised it critically for important intellectual content. All authors read and approved the final manuscript.
